# Perianal squamous cell carcinoma with high-grade anal intraepithelial neoplasia in an HIV-positive patient using highly active antiretroviral therapy: case report

**DOI:** 10.1590/S1516-31802007000500009

**Published:** 2007-09-02

**Authors:** Caio Sergio Rizkallah Nahas, Edesio Vieira da Silva, Jose Eduardo Levi, Fabio Cesar Atui, Carlos Frederico Sparapan Marques

**Keywords:** Carcinoma in situ, Anal cancer, Anus neoplasms, AIDS serodiagnosis, Human papillomavirus 16, Carcinoma in situ, Canal anal, Câncer do ânus, Sorodiagnóstico da aids, Papillomavirus humano 16

## Abstract

**CONTEXT:**

Highly active antiretroviral therapy (HAART) has turned human immunodeficiency virus (HIV) infection into a chronic condition, and this has led to increased incidence of anal dysplasia among HIV-positive patients. Routine anal evaluation including the anal canal and perianal area is recommended for this population, especially for patients infected by oncogenic human papillomavirus (HPV) types.

**CASE REPORT:**

A 54-year-old homosexual HIV-positive man presented with a six-year history of recurrent perianal and anal warts. He had previously undergone incomplete surgical excision and fulguration in another institution on two occasions. He had been using HAART over the past two years. He presented some condylomatous spreading lesions occupying part of the anal canal and the perianal skin, and also a well-demarcated slightly painful perianal plaque of dimensions 1.0 x 1.0 cm. Both anal canal Pap smears and biopsies guided by high-resolution anoscopy revealed high-grade squamous intraepithelial lesion. Biopsies of the border of the perianal plaque also revealed high-grade squamous intraepithelial lesion. HPV DNA testing of the anus detected the presence of HPV-16 type. The patient underwent local full-thickness excision of the lesion. Histological analysis on the excised tissue revealed high-grade squamous intraepithelial lesion with one focus of microinvasive squamous cell cancer measuring 1 mm. No lymph vessel or perineural invasion was detected. The patient showed pathological evidence of recurrent anal and perianal high-grade squamous intraepithelial lesions at the sixth-month follow-up and required further ablation of those lesions. However no invasive squamous cell carcinoma recurrence has been detected so far.

## RESUMO

**CONTEXTO:**

A terapia anti-retroviral altamente ativa tornou a infecção pelo HIV uma condição crônica, levando a um aumento na incidência de pacientes HIV-positivos com displasia anal. A avaliação anal rotineira incluindo a região anal e perianal é recomendada nessa população, especialmente nos doentes infectados por tipos oncogênicos do HPV.

**RELATO DE CASO:**

Um homem homossexual HIV-positivo de 54 anos foi encaminhado para avaliação de condilomas anais e perianais recorrentes há seis anos. Referia duas ressecções cirúrgicas incompletas em outro serviço. Encontrava-se em uso de medicação anti-retroviral altamente ativa há dois anos. Ao exame proctológico, apresentava algumas lesões condilomatosas no canal anal e na região perianal, assim como uma placa bem delimitada de 1,0 por 1,0 cm levemente dolorosa na região perianal. Ambos esfregaço do canal anal e biópsia guiada por anoscopia de alta resolução revelaram lesão escamosa intraepitelial de alto grau. Uma biópsia da borda da placa perianal também diagnosticou lesão escamosa intraepitelial de alto grau. A pesquisa de DNA de HPV do canal anal detectou a presença do HPV tipo 16. O paciente foi submetido à excisão local profunda da lesão. O estudo anatomopatológico revelou lesão escamosa intra-epitelial de alto grau com um foco de 1 mm de carcinoma epidermóide microinvasivo, sem invasão perineural ou linfo-vascular. O paciente apresentou recorrência de lesão escamosa intra-epitelial de alto grau no canal anal e na região perianal confirmadas por biópsias no sexto mês pós-operatório, necessitando ablação. Entretanto, até o momento não apresentou recorrência do carcinoma epidermóide invasivo.

## INTRODUCTION

Anogenital condyloma acuminata are benign proliferative lesions caused by human papillomavirus (HPV). Many subtypes of HPV have been identified. Some of them, particularly HPV 16, can lead to premalignant transformation of squamous epithelial cells, resulting in high-grade squamous intraepithelial lesions or even invasive squamous cell carcinoma.^1^

Diagnosing squamous intraepithelial lesions requires pathological examination. Cytological (anal Pap smears) and histological examination by biopsy under high-resolution anoscopy guidance are useful for internal evaluation of the anus. However, the perianal region, which is also susceptible to malignancy, is not routinely evaluated.

Here, we report on the case of a human immunodeficiency virus (HIV)-positive man who was referred to our service for evaluation of anal warts and presented with perianal microinvasive squamous cell carcinoma.

## CASE REPORT

**Figure 1:**
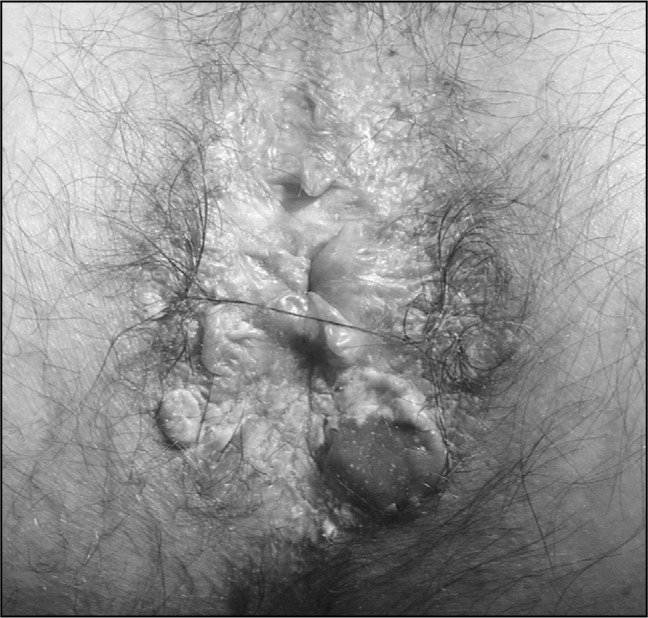
Perianal area: preoperative appearance.

A 54-year-old homosexual male presented to us with a six-year history of recurrent anal warts. He had previously undergone incomplete surgical excision and fulguration of anal and perianal warts in another institution on two occasions. He had tested positive for HIV six years earlier and had been well controlled with highly active antiretroviral therapy (HAART) over the past two years. His CD4 lymphocyte count was 253/mm^3^ and he had an undetectable HIV viral load. He presented some condylomatous lesions occupying part of the anal canal and the perianal skin, and also a well-demarcated slightly painful perianal erythematous plaque of dimensions 1.0 x 1.0 cm, with an irregular border and surface (Figure 1). Serological tests for herpes simplex virus and syphilis were negative.

**Figure 2:**
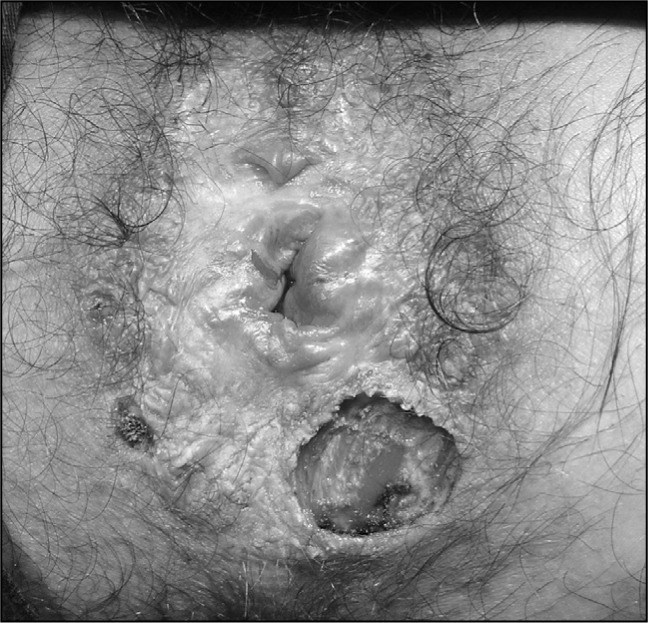
Perianal area: postoperative appearance.

The patient's anal Pap smear revealed high-grade squamous intraepithelial lesion and this was confirmed by anal biopsy guided by 3% acetic acid using high-resolution anoscopy. Biopsies were also performed at the border of the suspicious perianal plaque. HPV DNA testing was conducted using a well-established MY09/MY11 polymerase chain reaction assay and HPV-16 type was detected. Pathological examination of the perianal lesion biopsies revealed high-grade squamous intraepithelial lesion. Because of suspected malignancy, we performed local full-thickness excision of the lesion (Figure 2), in addition to ablation of the remaining condyloma. The wound was allowed to heal by secondary intention.

Histological analysis on the excised tissue revealed high-grade squamous intraepithelial lesion with one focus of microinvasive squamous cell carcinoma measuring 1 mm. No lymph vessel or perineural invasion was detected. Despite good postoperative recovery, the patient showed pathological evidence of recurrent anal and perianal high-grade squamous intraepithelial lesions, which were detected by high-resolution anoscopy at the sixth-month follow-up and required further ablation of those lesions. However, no cancer recurrence has been detected so far.

## DISCUSSION

Anal cancer shares many biological characteristics with cervical cancer, including similar histopathological appearance and association with HPV infection. Recent studies have shown that both anal squamous intraepithelial lesions and anal HPV infection are more common in HIV-positive homosexual or bisexual men than in HIV-negative men, which suggests that HIV infection may even increase the risk of developing anal cancer.^2^

Although most of the research on anogenital neoplasia in HIV-infected individuals has focused on lesions of the cervix and anal canal, there is also evidence that similar lesions occur with increased frequency in perianal skin (5 cm radially from the anal verge).^3^ These lesions are often multifocal and frequently recur after standard treatments. It has been proposed that populations at high risk of developing squamous cell carcinoma of the anus should be screened regularly by means of anal cytology in combination with high-resolution anoscopy.^3,4^ The perianal area should be routinely examined for suspicious lesions, since perianal dysplasia may present with macroscopic or microscopic lesions. Symptomatic patients complain of perianal bleeding, pruritus, burning and anal discharge, and may present irregular brownish-red, eczematoid lesions. Paucity of symptoms and slow growth often result in delayed diagnosis.

There is a lack of literature regarding the incidence of specifically perianal dysplasia in HIV-infected patients. However, the incidence of anal dysplasia has been well described. In a study covering a four-year period, Palefsky et al.^5^ observed that HIV-positive men were more likely to develop anal high-grade squamous intraepithelial lesions than were HIV-negative men (relative risk 3.7; 95% confidence interval, CI: 2.6-5.7). High rates of anal high-grade squamous intraepithelial lesions among HIV-positive men, at all CD4 levels but with lower CD4 counts, have been associated with earlier development of high-grade squamous intraepithelial lesions (p = 0.007).

Multiple oncogenic HPV-type infections are very common among HIV-positive homosexual men and show almost a 100% association with anal squamous cell carcinoma, especially HPV type 16.^6^ Although it is well established that HIV seropositivity and low CD4 T-cell count are positively associated with the overall prevalence of HPV,^6^ few data have been reported regarding these associations on an individual HPV type-specific basis.

Surgical excision is considered to be the therapy of choice for perianal dysplasia and can be safely performed, especially for patients with limited disease like in our case. However, for more extensive or diffuse perianal high-grade squamous intraepithelial lesions, treatment can be challenging. Alternative treatments for the latter situation includes cryosurgery, 5-fluorouracil cream, photodynamic therapy and imiquimod 5% cream. Wide local excision with free uninvolved margins may leave big reconstructive challenges like extensive flaps or grafts and the risk of incontinence. However, in such cases, conservative management seems to be favored by most surgeons, according to a survey of members of the American Society of Colon and Rectal Surgeons.^7^ It may not be possible to eliminate the virus, and therefore the goal of treatment is to eliminate macroscopic lesions that may be more likely to progress to invasive carcinoma. Chemoradiation is reserved for patients with invasive squamous cell carcinoma.

In the current case, excisional biopsy was useful for diagnosing and treating very early-stage cancer. Since there was no deep invasive squamous cell carcinoma, we considered it appropriate not to offer adjuvant treatment.

Our patient illustrates a complication for HIV-positive patients that is associated with prolonged survival attributed to HAART. Immune status improvement has effectively decreased some viral infections and malignancies such as molluscum contagiosum and Kaposi's sarcoma. However, HPV infections and associated malignancies have not responded as favorably.^8–10^ Thus, routine examination of the anal canal in this population is recommended, as described in a screening algorithm.^3^ Additionally, we recommend careful examination of the perianal skin to rule out malignancies in all HIV-positive patients, especially those who have tested positive for oncogenic anogenital HPV infection.

## References

[1] Beckmann AM, Daling JR, Sherman KJ (1989). Human papillomavirus infection and anal cancer. Int J Cancer.

[2] Melbye M, Palefsky J, Gonzales J (1990). Immune status as a determinant of human papillomavirus detection and its association with anal epithelial abnormalities. Int J Cancer.

[3] Goldstone SE, Winkler B, Ufford LJ, Alt E, Palefsky JM (2001). High prevalence of anal squamous intraepithelial lesions and squamous-cell carcinoma in men who have sex with men as seen in a surgical practice. Dis Colon Rectum.

[4] Jay N, Berry JM, Hogeboom CJ, Holly EA, Darragh TM, Palefsky JM (1997). Colposcopic appearance of anal squamous intraepithelial lesions: relationship to histopathology. Dis Colon Rectum.

[5] Palefsky JM, Holly EA, Ralston ML, Jay N, Berry JM, Darragh TM (1998). High incidence of anal high-grade squamous intra-epithelial lesions among HIV-positive and HIV-negative homosexual and bisexual men. AIDS.

[6] Frisch M, Biggar RJ, Goedert JJ (2000). Human papillomavirus-associated cancers in patients with human immunodeficiency virus infection and acquired immunodeficiency syndrome. J Natl Cancer Inst.

[7] Cleary RK, Schaldenbrand JD, Fowler JJ, Schuler JM, Lampman RM (1999). Perianal Bowen's disease and anal intraepithelial neoplasia: review of the literature. Dis Colon Rectum.

[8] Palefsky JM, Holly EA, Efirdc JT, Da Costa M, Jay N, Berry JM, Darragh TM (2005). Anal intraepithelial neoplasia in the highly active antiretroviral therapy era among HIV-positive men who have sex with men. AIDS.

[9] Heard I, Palefsky JM, Kazatchkine MD (2004). The impact of HIV antiviral therapy on human papillomavirus (HPV) infections and HPV-related diseases. Antivir Ther.

[10] Palefsky JM, Holly EA, Ralston ML (2001). Effect of highly active antiretroviral therapy on the natural history of anal squamous intraepithelial lesions and anal human papillomavirus infection. J Acquir Immune Defic Syndr.

